# A network analysis of gene co-expression in post-mortem brain tissues identifying novel genes and biological pathways underlying major depression

**DOI:** 10.3389/fpsyt.2025.1556983

**Published:** 2025-07-11

**Authors:** Miaomiao Yan, Man Chen, Qin Jiang, Huilan Huang, Yigao Wu

**Affiliations:** ^1^ Department of Nursing, The First Affiliated Hospital of Wannan Medical College, Wuhu, China; ^2^ Department of Medical Psychology, The First Affiliated Hospital of Wannan Medical College, Wuhu, China

**Keywords:** major depressive disorder, bipolar disorder, schizophrenia, oxidative phosphorylation of mitochondria, ubiquitination

## Abstract

**Introduction:**

Major Depressive Disorder (MDD), also known simply as depression, is a serious and common mood disorder. However, the potential pathogenesis and target genes of the disease have not been well illustrated.

**Methods:**

We identified differentially expressed genes(DEG) using the published transcriptome data GSE80655 from postmortem brain tissues of 69 MDD patients, 71 Bipolar Disorder (BD) patients, and 71 Schizophrenia(SZ) patients and 70 controls. Another brain expression dataset included geneexpression profiles from six cortical and limbic brain regions of 34 patients with MDD and 55 control subjects without any history of psychiatric or neurological disorders. GO enrichment analysis was performed on DEGs.Weighted gene Co-expression network analysis (WGCNA) was conducted on DEGs. Subsequently,we extracted the modules determined by WGCNA and calculated the correlation of the modules with diseases or brain tissue regions. The modules specifically related to MDD were screened. Module genes are employed for gene function enrichment analysis.Enriched genes in biological pathways are used to construct the gene regulatory network, and the genes with the highest connectivity are classified as hub genes.

**Results:**

DEGs related to MDD, BD, or SZ were screened. And by WGCNA, we determined the gene modules associated with MDD but not with BD and SZ. The MDD-related modules were further compared with the gene expression data from postmortem brain tissue of MDD patients in another group, and one of the gene modules which was enriched in the biological pathway related to mitochondrial oxidative phosphorylation and ubiquitination, was screened.Gene regulatory network analysis showed that the hub genes were PARK2, CUL1, SKP1, CYC1, and ATP5A1.The mitochondrial oxidative phosphorylation and ubiquitination related biological pathways are involved in the process of MDD.

**Discussion:**

The hub genes may provide possible candidates for MDD targeted therapy that is worth exploring.

## Introduction

Major depressive disorder (MDD) is one of the leading causes of disability worldwide, which affects about 322 million people and may lead to disability (1). This disease has a complex cause because it is affected by various environmental conditions (such as life experience) and genetic differences. A study on twins with depression showed that genetic factors accounted for about 37% of the contribution (2). Genome-wide association studies (GWASs) also suggested that common single-nucleotide polymorphisms (SNPs) contributed 9% to MDD. These results suggest that the occurrence and development of MDD are related to genetic factors (3). Wray et al. found 44 MDD-associated SNP sites in previous GWASs (*n* = 135,458 cases) (4). Howard et al. found 102 MDD-associated SNP sites using a larger sample (*n* = 246,363 cases) and a broader phenotype definition (3). Although genes associated with susceptibility to MDD can be found by GWASs, the changes in gene function caused by genetic variation have not been confirmed. Therefore, exploring the changes in gene expression and involved biological pathways in MDD patients may provide more clues on the molecular mechanisms driving depression.

So far, previous studies discovered the relationship between gene expression and depression (4–7). It was demonstrated by Wray et al. that 17 genes (including OLFM4, PXDNL, NDUFA2, etc.) in the dorsolateral prefrontal cortex (DLPFC) had association with MDD (4). Gaspar et al. found that the significant up- and downregulation of 153 genes in the brain region also had a close link to MDD, of which 24 genes were drug targets (7). Based on gene network, an analysis for gene expression data of multiple brain tissues and whole blood was carried out by Gerring et al. It revealed 99 biologically possible risk genes related to MDD, 58 of which were new, and identified 11 (cortex) and 24 (amygdala) mutually exclusive modules, indicating the relationship between the heterogeneity of different brain regions and MDD (5). A study conducted by Gamazon et al. determined gene expression changes related to various psychiatric diseases in a variety of tissues, showing that different psychiatric diseases have unique gene expression patterns (6). In addition, nucleus accumbens (nAcc) regions, DLPFC and anterior cingulate cortex (AnCg), are usually related to emotional change, cognition, reward, motivation, impulse control, and happiness (8, 9). These factors may be involved in affecting MDD, so probing into the relationship between gene expression changes and MDD is required. In order to find these related genes, by utilizing the transcriptome data of postmortem brain tissue, this study analyzed the gene expression changes in the AnCg, DLPFC, and nAc regions of MDD, bipolar disorder (BD), and schizophrenia (SZ) patients, respectively. Besides that, gene co-expression modules were constructed to find MDD-specific gene modules and finally screened out MDD-specific gene changes. These results are helpful for understanding the biological mechanism of depression. The comparison with other mental diseases highlights the key genes of MDD. These genes may be useful candidates for future research on its etiology and therapeutic targets.

## Materials and methods

### Data collection and preprocessing

From the Gene Expression Omnibus (GEO) (10), we obtained transcriptome data (GSE80655) of 281 clinically described human postmortem brain tissues, including AnCg, nAcc, and DLPFC of the brain as well as samples of MDD (*n* = 69), BD (*n* = 71), SZ (*n* = 71), and controls (*n* = 70). MDD is diagnosed based on persistent low mood, loss of interest, fatigue, changes in appetite, sleep disturbances, and thoughts of death lasting for at least 2 weeks. BD is identified by extreme mood swings, including manic or hypomanic episodes with elevated mood and depressive episodes. Diagnosis requires at least one manic or hypomanic episode. SZ is diagnosed with symptoms like hallucinations, delusions, and disorganized thinking, lasting for at least 6 months, with 1 month of active-phase symptoms. Each disorder has distinct mood or psychotic features but can share overlapping symptoms. Transcriptome sequencing for the dataset was performed on the Illumina HiSeq 2000 platform with 50× coverage and 100-bp read length. The pooled libraries were sequenced on an Illumina HiSeq 2000 sequencing machine using paired-end 50-bp reads and a 6-bp index read, resulting in an average of 48.2 million reads per library. We performed an analysis of DEGs in GEO database in GEO2R platform and obtained the statistical results in the form of a table. Another brain expression data for MDD was derived from a study by Jun Z. Li et al. (11), which included 55 subjects with no history of psychiatric or neurological disease (as a control group) and six cortical and limbic brain regions in 34 MDD patients. The gene expression data from the dataset of Jun Z. Li et al. were used for the cross-validation of MDD-specific modules identified via WGCNA in the primary dataset. Specifically, we examined whether genes from the MDD-specific modules (particularly the “midnightblue” module) showed consistent expression trends in the six cortical and limbic regions in this independent dataset.

### Identify differentially expressed genes in MDD

For the expression data of three brain regions, the “limma” R software package was used to determine the genes with a significant differential expression between the MDD and control groups. The *p*-values were adjusted using the Bonferroni correction method, with threshold log2FC >0 or <0 and *p*-value <0.05.

### Construction of weighted gene co-expression networks and identification of modules associated with MDD and brain regions

We performed gene co-expression network analysis exclusively on brain tissues using the R software WGCNA. The soft-thresholding power (β) is chosen to ensure a scale-free topology, typically between 6 and 20. The minimum module size sets the smallest number of genes in a module, usually around 30. The merge cut height is the threshold for merging similar modules, often set between 0.25 and 0.30. TOM (topological overlap matrix) is used to measure gene similarity, and hierarchical clustering identifies co-expressed genes. Finally, module–trait relationships assess the correlation between modules and external traits. The expression matrix is limited to the differentially expressed genes of MDD. Gene expression was normalized to counts per million (CPM) and log-transformed (log10 (CPM+1)). Then, we determined the optimal soft threshold for adjacency computation of gene co-expression networks in graphical form (**Supplementary Figure S1**). The transformed expression matrix was input into the function modules of the WGCNA software package, and the corresponding eigengenes were obtained. The cutreeDynamic function was applied to prune the gene hierarchical clustering tree, resulting in color-labeled co-expression modules; the related modules were then merged (*r* > 0.75).

The dissimilarity of module eigengenes (ME) was calculated with the module eigengenes function in the WGCNA package. The relationship between the eigengenes value with three brain regions, MDD, BD, and SZ was evaluated by using Pearson’s correlation.

### Construction of protein–protein interaction networks of selected modules and identification of hub genes

First, the association between the genes in the module and module eigengenes was estimated by using Pearson’s correlation, which was called module membership (MM). Hub genes were selected as genes with MM >0.8 in specific modules. Subsequently, we constructed a connection network by using the gene regulatory relationship based on the STRING database (https://cn.string-db.org/) and selected the core genes according to the degree of connection. The parameters include interaction score thresholds (a minimum confidence score of 0.4 to 0.9) to filter interactions, evidence types (experimental data, text mining, and co-expression), and species selection to focus on interactions specific to the organism of interest. The users can also adjust network display settings, such as the network node size and coloring based on different attributes (gene expression levels).

### GO enrichment analysis

GO enrichment analysis of differentially expressed genes was conducted using Metascape (https://metascape.org/gp/index.html). The enrichment parameters for pathways and processes were configured as follows: minimum overlap of 3, *p*-value cutoff of 0.01, and minimum enrichment of 1.5. Terms with a *p*-value <0.01 were considered significantly enriched in GO terms. The enriched terms were chosen based on the cluster results from Metascape.

## Result

### Differential gene expression related to MDD

**Figure 1:**
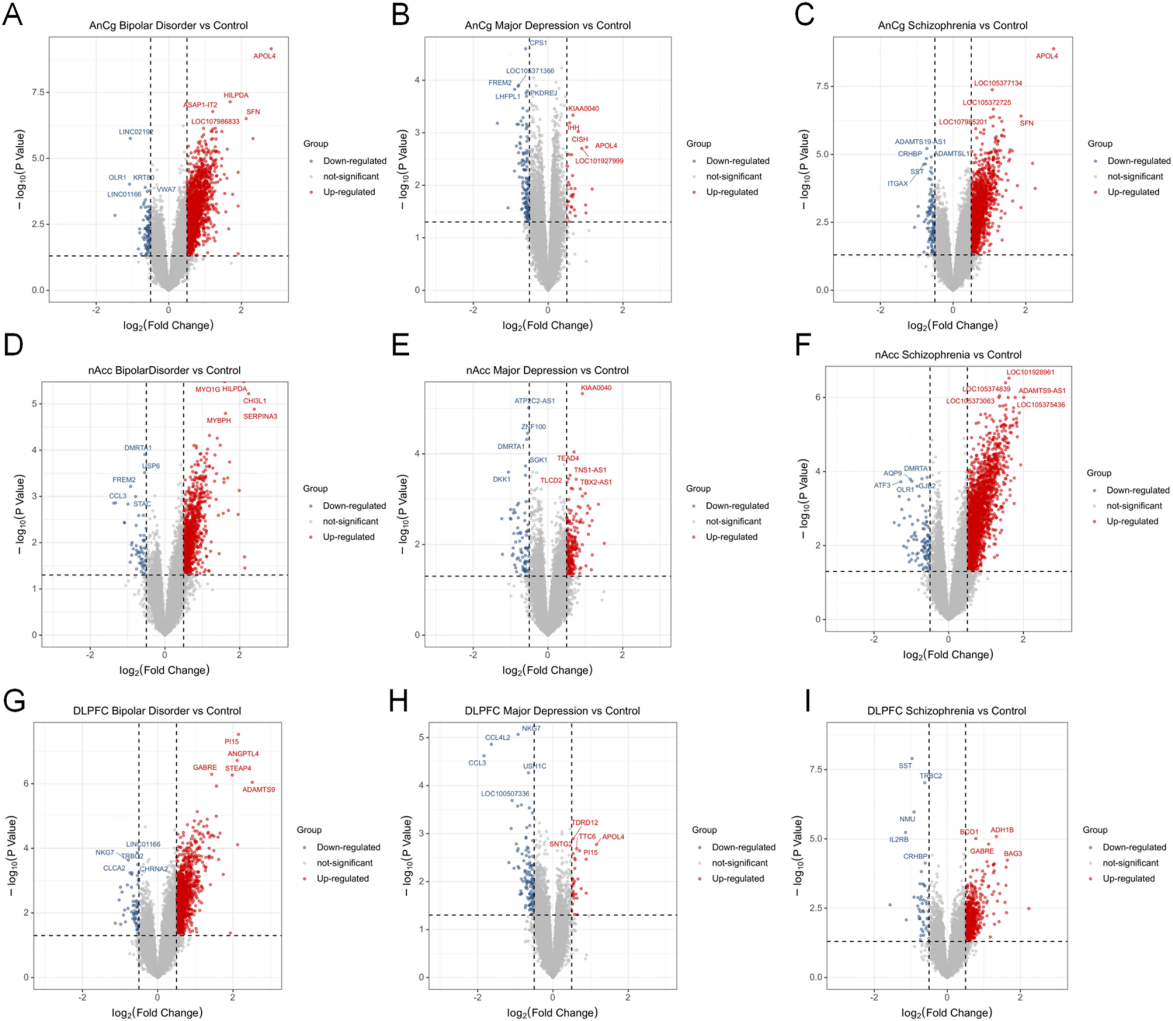
Differentially expressed genes of MDD patients, bipolar disorder (BD) patients, and schizophrenia (SZ) patients compared to the control group. Volcano plots show the multiple and *p*-value of gene expression changes in brain tissues of different diseases. **(A–C)** Differential genes at AnCg site. **(D–F)** Differential genes at **D–F** and nAcc site. **(G–I)** Differential genes at DLPFC site.

After collection of transcriptome data of human postmortem brain tissue, an expression difference analysis was carried out to compare the difference of AnCg, DLPFC, and nAcc samples of MDD, BD, and SZ patients with the control group. **Figure 1** shows the significant DEGs (log2FC >0 or <0 and *p*-value < 0.05) among MDD, BD, and SZ patients compared to the control group in the AnCg, DLPFC, and nAcc regions. The result showed that in the AnCg region, there were significantly upregulated genes 1,717, 3,452, and 2,997 in MDD, BD, and SZ patients, respectively, and significantly downregulated genes 916, 3,771, and 3,226 in MDD, BD, and SZ patients, respectively. In the nAc region, 798, 888, and 2,598 genes were remarkably elevated in MDD, BD, and SZ patients, respectively, while 635, 685, and 1,247 genes showed a marked drop in MDD, BD, and SZ patients, respectively. In the DLPFC region, 660, 1,378, and 841 genes were increased in MDD, BD, and SZ patients, respectively; 634, 1,377, and 956 genes were significantly reduced in MDD, BD, and SZ patients, respectively (**Figure 1**). Detailed gene expression information is provided in **Supplementary Table S1**. To validate our findings, we compared the MDD-related modules from GSE80655 with the gene expression patterns in the dataset of Jun Z. Li et al. Among the top 100 highly expressed genes across six brain regions, 34 overlapped with those in the “midnightblue” module, reinforcing its relevance to MDD.

### GO enrichment analysis of differentially expressed genes

**Figure 2:**
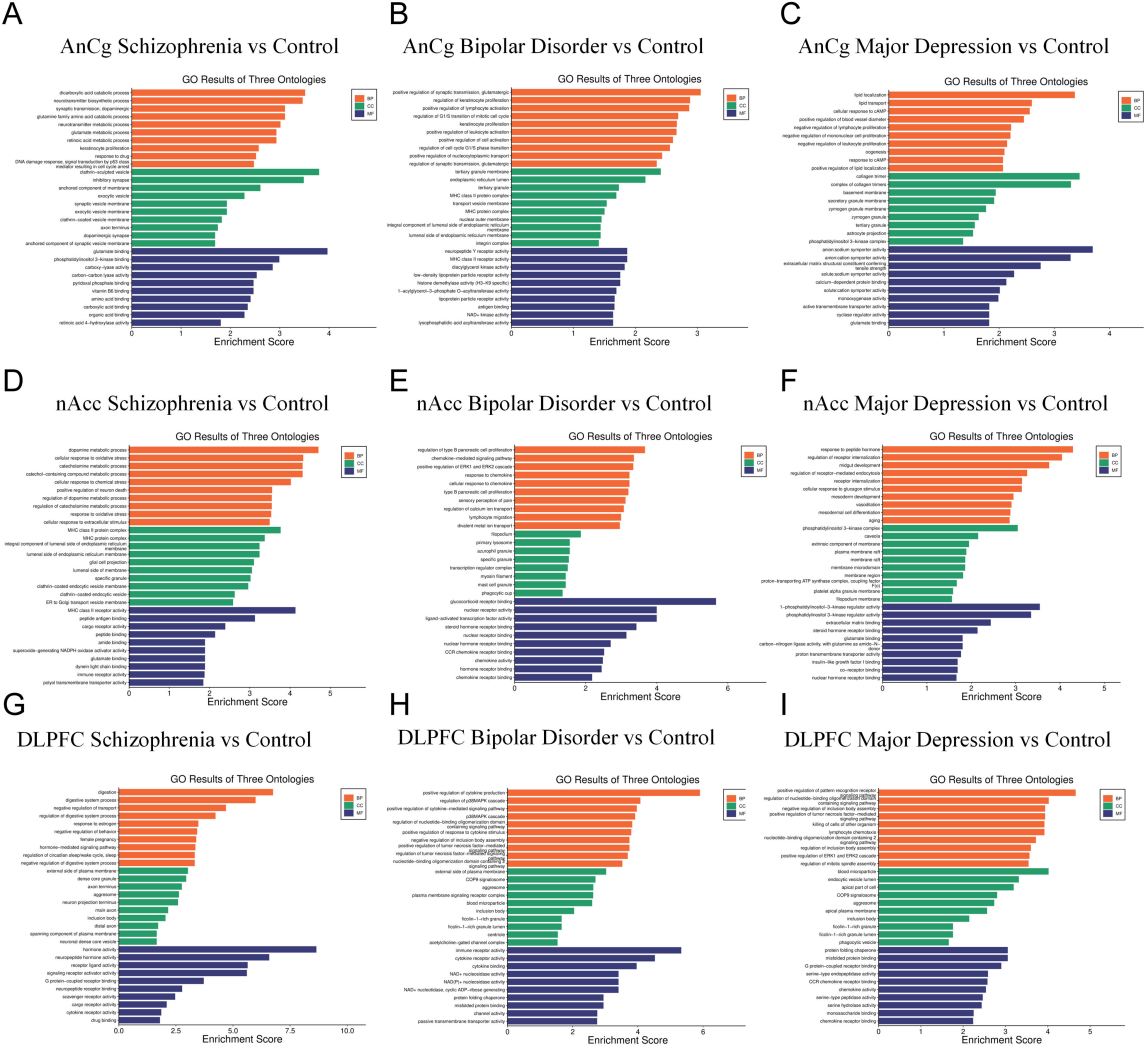
GO enrichment analysis of the top 20 differentially expressed genes of SZ, BD, and MDD patients compared to the control group. **(A–C)** GO enriched pathways at AnCg site. **(D–F)** GO enriched pathways at nAcc site. **(G–I)** GO enriched pathways at DLPFC site.

Next, we performed GO enrichment analysis on the top 20 differentially expressed genes from AnCg, nAcc, and DLPFC samples of SZ, BD, and MDD patients compared to the control group. The result showed that, in the AnCg region, dicarboxylic acid catabolic process, clathrin-sculpted vesicle, and glutamate binding were the top enriched pathways in SZ patients (**Figure 2A**), positive regulation of synaptic transmission, glutamatergic, tertiary granule membrane, and neuropeptide Y receptor activity were the top enriched pathways in BD patients (**Figure 2B**), and lipid localization, collagen trimer, and anion/sodium symporter activity were the top enriched pathways in MDD patients (**Figure 2C**). In the nAcc region, dopamine metabolic process, MHC class II protein complex, and MHC class II receptor activity were the top enriched pathways in SZ patients (**Figure 2D**), regulation of type B pancreatic cell proliferation, filopodium, and glucocorticoid receptor binding were the top enriched pathways in BD patients (**Figure 2E**), and response to peptide hormone, phosphatidylinositol 3-kinase complex, and 1-phosphatidylinositol-3-kinase regulator activity were the top enriched pathways in MDD patients (**Figure 2F**). In the DLPFC region, digestion, external side of plasma membrane, and hormone activity were the top enriched pathways in SZ patients (**Figure 2G**), positive regulation of cytokine production, external side of plasma membrane, and immune receptor activity were the top enriched pathways in BD patients (**Figure 2H**), positive regulation of pattern recognition receptor signaling pathway, blood microparticle, and protein folding chaperone were the top enriched pathways in MDD patients (**Figure 2I**).

### Co-expression network of MDD-related genes

**Figure 3:**
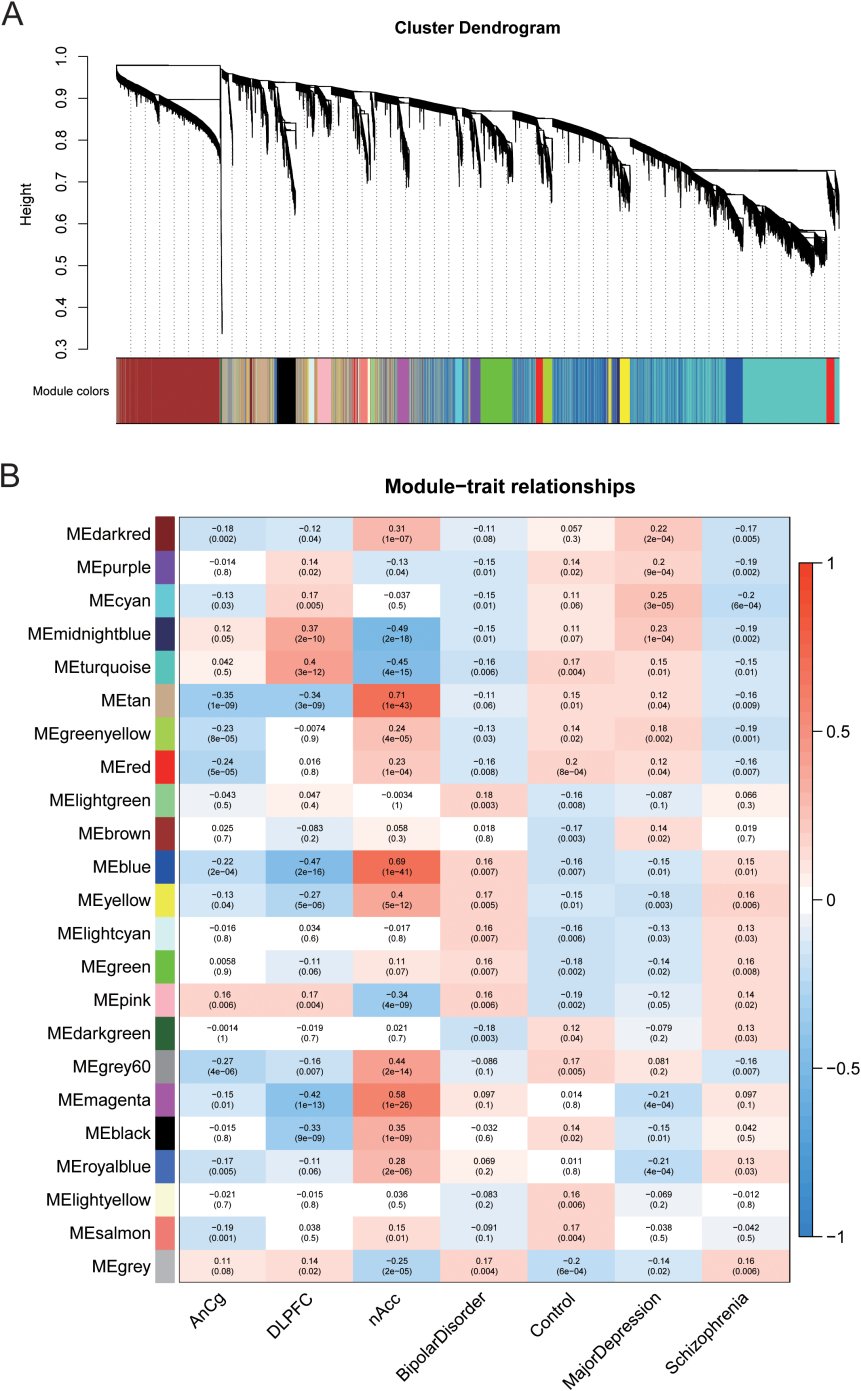
Different gene modules were obtained through weighted gene co-expression network analysis (WGCNA) on different genes. **(A)** Based on the weighted correlation, hierarchical clustering analysis was carried out, and the clustering results are segmented according to the set criteria to obtain different gene modules. **(B)** The heat map shows the correlation between the expression trend of each gene module and AnCg, DLPFC, and nAcc in MD, BD, and SZ.

In order to find MDD-related co-expressed gene modules and to explore the association between the gene network and MDD, BD, and SZ as well as the core genes in the network, WGCNA analysis was conducted. Based on the finding of the significantly different genes, we extracted each sample and the expression value of each significantly different gene and finally built a gene co expression network. Then, hierarchical clustering analysis was performed based on the weighted correlation, and the clustering results were segmented according to the set criteria to obtain different gene modules. Different modules are represented by the branches of the clustering tree and different colors. A total of 23 gene modules are found (**Figure 3A**). Genes in the same module have similar expression patterns, and subsequently the first principal component (MES, module eigengene) of each module is calculated to represent the gene expression profile of the whole model. Based on MEs data, we evaluated the correlation between the expression trend of each module and MDD, BD, SZ, AnCg, DLPFC, and nAccs (**Figure 3B**). The results demonstrated that MDD, BD, and SZ had their specific associated genes, among which the gene modules with high correlation with MDD were obviously different from the other two. The gene modules darkred, cyan, and midnightblue were correlated with MDD, significantly higher than those in the control group, and different from the other two diseases. The gene module darkred is specifically overexpressed in the nAcc region, while the gene modules cyan and midnightblue are highly expressed in the DLPFC region. The gene modules magenta and royalblue in MDD were significantly lower than those in the control group and highly expressed in the nAcc region, which were different from the other two diseases. Lastly, we calculated the correlation (MM, module membership) between the respective genes in each module and the module ME and screened the genes with MM greater than 0.8 as the core genes of each module for further gene screening.

### The MDD-related gene module midnightblue contained genes highly expressed in brain tissue

**Figure 4:**
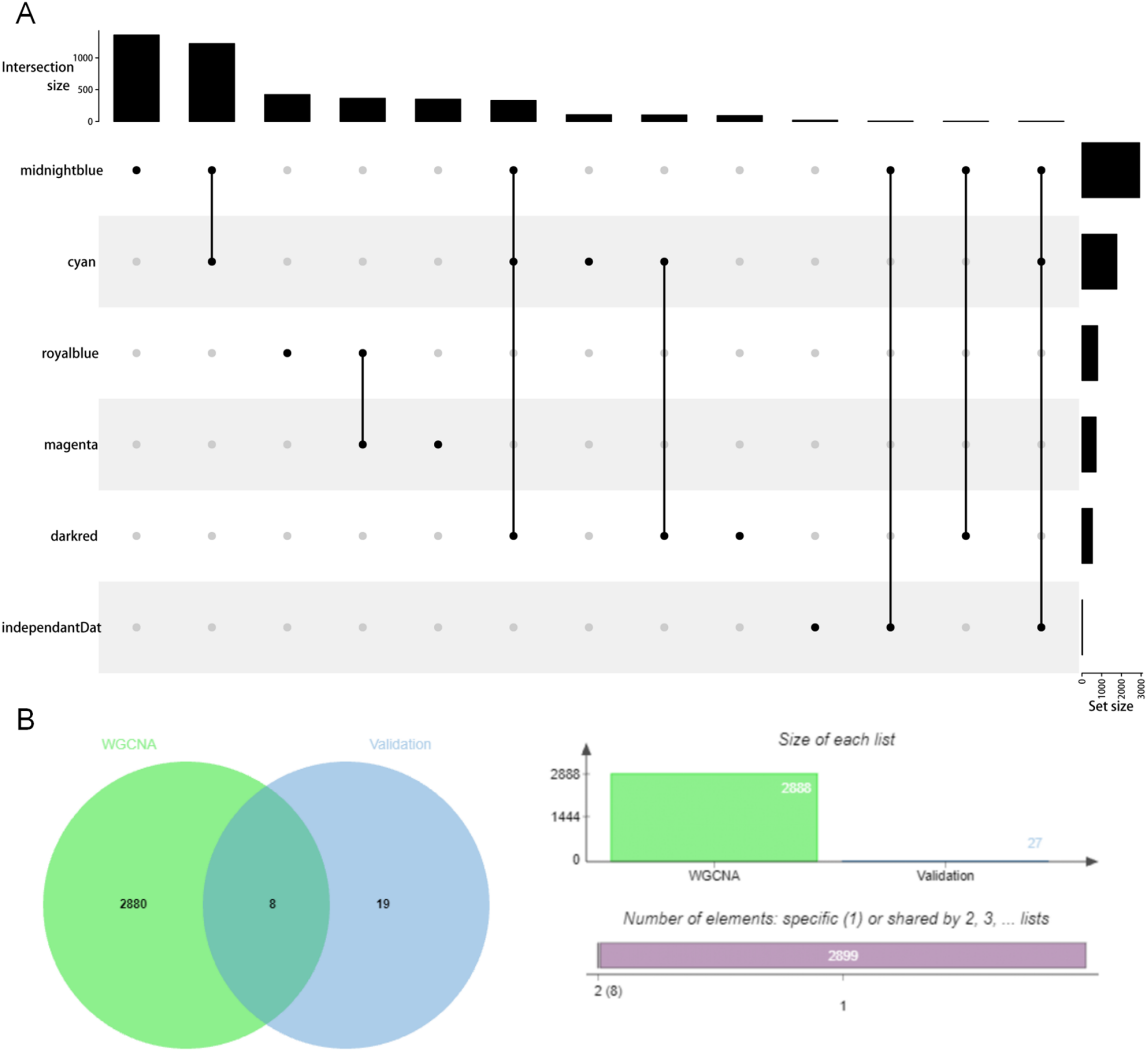
The overlapping genes in the MDD-related gene module and in the high expression gene module of MDD brain expression data.

To further determine the most MDD-related gene modules, we found another transcriptome data set of human postmortem brain tissue to obtain highly expressed genes in the brain tissue of MDD patients. This data set contained the gene expression data of six brain regions of MDD patients (DLPFC, nAcc, AnCg, HC, CB, and Amy) and the gene expression data of the controls. A total of 34 genes—significant differential genes related to MDD—were found in the top 100 genes with high expression in each region, and they were also existing in midnightblue compared to darkred and cyan (**Figure 4**), suggesting that midnightblue has genes that are highly expressed in brain tissue and has a high correlation with MDD.

### Gene function in the MDD-related gene module midnightblue

**Figure 5:**
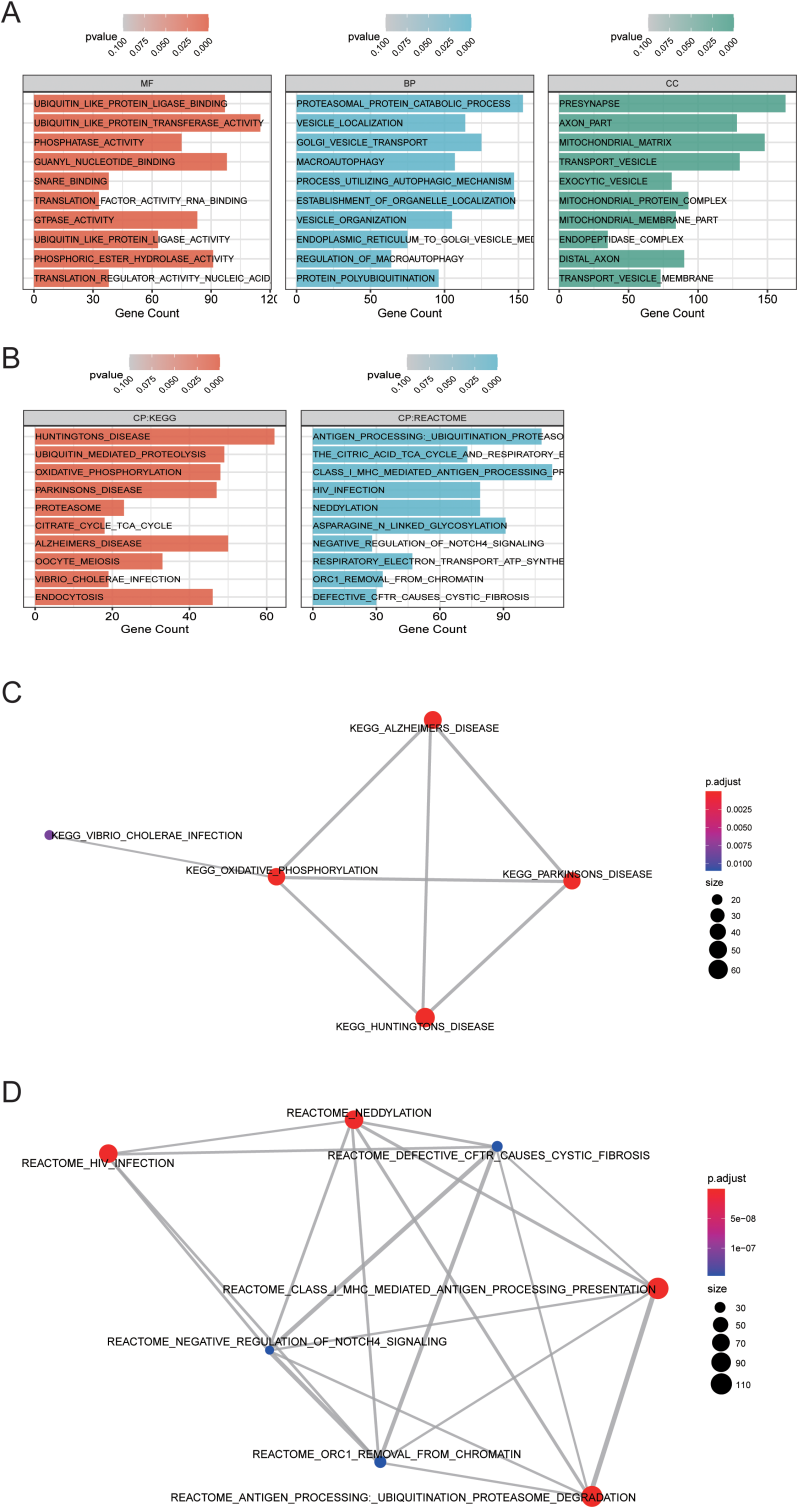
Function enrichment analysis in MDD-related gene module midnightblue. **(A)** Gene oncology enrichment analysis results. **(B)** KEGG path enrichment analysis results. **(C, D)** The overlapping relationship was analyzed between the enriched biological functions and pathways, and a connection network between them was built.

Based on Gene Ontology (GO) database, the genes in midnightblue are annotated from BP, MF, and CC levels. Fisher test was used to calculate the significance level (*P*-value) of each GO, so as to screen the GO term (**Figure 5A**) with rich gene significance. Simultaneously, the genes in midnightblue were annotated based on KEGG database to get all the pathway terms involved in the gene. Pathway terms with rich gene significance (**Figure 5B**) were screened out by calculating the significance level (*P*-value) of the pathway via Fisher test. Then, we analyzed the overlapping relationship of these enriched biological functions and pathways and built a connection network between them (**Figures 5C, D**). Through enrichment analysis, we obtained the functional enrichment of genes in the midnightblue module. Through the results of the pathway terms relationship network, it can be inferred that the genes related to oxidative phosphorylation and the genes in class I mhc-mediated antigen processing presentation play critical roles in MDD, which all overlap with related genes of neurological diseases (Alzheimer’s disease, Parkinson’s disease, and Huntington disease) (12). The genes in oxidative phosphorylation pathway are basically the genes of mitochondrial oxidative phosphorylase. Most of the genes in class I mhc-mediated antigen processing presentation are the substrates of E3 ubiquitin ligase and participate in ubiquitination. These findings revealed that linear metabolism and ubiquitination were correlative to MDD.

**Figure 6:**
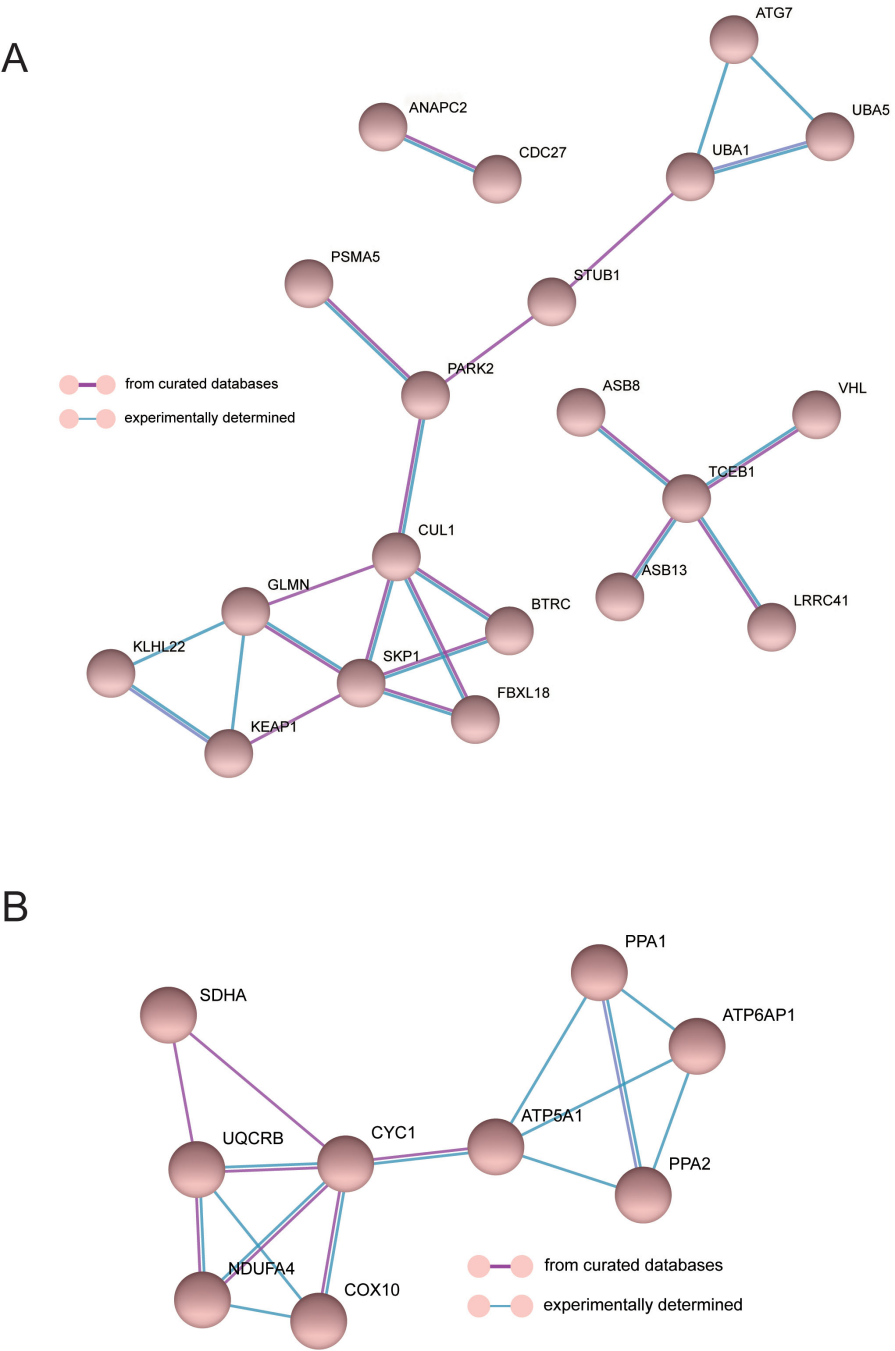
The interaction network of MDD genes in the oxidative phosphorylation **(A)** and class I mhc-mediated antigen processing presentation pathways **(B)**.

To investigate the interaction between genes and their mechanism, we constructed the gene interaction network in oxidative phosphorylation and class I mhc-mediated antigen processing presentation pathways, respectively (**Figures 6A, B**), according to the gene regulation relationship recorded in STRING database and the signal transduction relationship between genes. Gene interaction network was used to analyze the direct or indirect interaction between genes by analyzing known signal pathways so as to understand the context of gene interaction and find core genes. Core gene is an important hub of gene interaction and plays a considerable role in network modules. PARK2, CUL1, SKP1, and TCEB1 are at the core of the connection in the network of class I mhc-mediated antigen processing presentation. CYC1 and ATP5A1 are at the core of the connection in the network of oxidative phosphorylation. These core genes may exert significant regulatory function in MDD.

## Discussion

In this study, we used postmortem brain tissue samples to systematically study the gene expression patterns and related differentially expressed genes in different brain tissue regions of three psychiatric diseases, SZ, BD, and MDD, and determined the common and unique gene modules related to these three types of mental diseases in brain tissue regions through WGCNA analysis. SZ and BD had many common related gene modules, but the expression mode of MDD is different from them. The analysis of gene expression in BD and SZ patients revealed distinct gene modules that were associated with these disorders when compared to the control group. In BD, genes in the AnCg, nAcc, and DLPFC regions exhibited both upregulated and downregulated expression patterns, which were different from those seen in MDD. Notably, the gene module cyan, highly expressed in the DLPFC, showed significant alterations, pointing to region-specific changes in BD. In SZ, similar patterns were observed, with genes in the nAcc and DLPFC regions showing marked upregulation and downregulation, respectively.

The GO enrichment analysis of the top 20 differentially expressed genes across AnCg, nAcc, and DLPFC samples from SZ, BD, and MDD patients revealed distinct biological processes and pathways associated with each disorder. In the AnCg region, SZ patients showed significant enrichment in pathways related to dicarboxylic acid catabolism and glutamate binding, which may reflect altered metabolic and neurotransmitter signaling in this disorder. BD patients, on the other hand, had enriched pathways related to synaptic transmission and neuropeptide Y receptor activity, suggesting a role of synaptic plasticity and neuropeptide modulation in the pathophysiology of BD. MDD patients exhibited enrichment in lipid localization and collagen trimer pathways, potentially pointing to disruptions in lipid metabolism and extracellular matrix components.

In the nAcc region, SZ patients showed enrichment in pathways related to dopamine metabolism, indicating a disruption in dopaminergic signaling, while BD patients had pathways related to cell proliferation and glucocorticoid receptor binding, which may reflect stress response and neuroplasticity mechanisms in BD. MDD patients showed enrichment in pathways related to hormone response and PI3K signaling, suggesting that hormonal and cell survival signaling could play a role in MDD. In the DLPFC region, the enriched pathways in SZ were related to digestion and hormone activity, which could indicate autonomic dysfunction, while BD and MDD patients exhibited pathways tied to immune response and cytokine production, highlighting potential immune system involvement in mood disorders. Overall, these findings emphasize the complex and region-specific molecular alterations in each psychiatric disorder, providing insights into their underlying pathophysiological mechanisms.

The gene modules darkred and midnightblue, correlated with SZ, were distinct from MDD and BD, indicating that SZ has unique molecular signatures. Additionally, the comparison of MDD with BD and SZ highlights the differences in gene expression patterns between these conditions, suggesting that although they share some common regions of dysregulation (such as the nAcc and DLPFC regions), they have specific gene modules that contribute to the distinct pathophysiology of each disorder. These findings suggest that BD and SZ have unique molecular signatures that could help in understanding the underlying biological mechanisms and aid in the development of targeted therapies. Finally, we screened the unique gene module midnightblue of MDD which was different from the other two diseases and mainly expressed in the DLPFC region. Subsequent gene function analysis indicated that the genes in midnightblue were mainly enriched in mitochondrial genes of oxidative phosphorylation and antigen presentation pathway regulated by E3 ligase. This indicated that these pathways and genes involved were related to MDD.

Through the network analysis of gene interaction, the regulatory genes related to MDD were further discovered. It was found that PARK2 can label CyclinE and other proteins with ubiquitin to guide protease to degrade them. When this gene mutates, it will not be able to perform the function of ubiquitination normally, leading to the aggregation of some cyclins and other functional proteins. It may cause abnormal proliferation in cells with mitotic conditions and apoptosis in neurons without mitosis (13). Similarly, CUL1, SKP1, and TCEB1 constitute E3 ubiquitination ligase and also perform the function of ubiquitination. These genes have not been reported to be associated with MDD. However, ubiquitination is involved in many biological processes and is related to some pathophysiological changes. At present, some studies also demonstrate that the change of ubiquitination is indeed associated with MDD. It is reported that inhibition of HSP90 ubiquitination would result in increased activation of microglia and participate in the occurrence of MDD (14). A GWAS study in a Japanese population shows that ubiquitin-specific 46 gene is related to the pathophysiology of MDD (15).

In addition, we found gene enrichment of mitochondrial oxidative phosphorylation in MDD-specific gene modules and identified core genes CYC1 and ATP5A1. Similarly, Liang et al. (16) also discovered the core genes related to Alzheimer’s disease (AD) and sleep disorders via gene module analysis, including ATP5A1, UQCRC2, ATP5B, UQCRC1, COX5A, SOD1, GAPDH, NDUFV2, NDUFA9, and NDUFS3 genes. Mitochondrial dysfunction is believed to be involved in the etiology and pathological process of MDD, and the change of oxidative phosphorylation system is the basis of the pathophysiological mechanism of MDD (17). Brain bioenergy abnormalities are often observed in adults with MDD because the regulation of high-energy phosphate metabolism and oxidative phosphorylation in MDD patients has changed, and the degree of oxidative phosphorylation is positively correlated with the severity of depression (18). The mitochondrial oxidative phosphorylated ATP5A1 gene is involved in the regulation of astrocyte maturation and synaptic formation (19), and synaptic damage affects neurodevelopment and mental diseases. CYC1 and ATP5A1, as the housekeeping genes, theoretically have little expression change under different physiological conditions. Nevertheless, the differential changes of these two genes in MDD patients were seen, which were unique to MDD patients (different from BD and SZ). These gene changes may not be the cause of MDD but may be the result of patients taking antidepressants because it is reported that antidepressants will affect the expression of housekeeping genes (20). Antidepressant treatments can significantly impact gene expression in MDD, potentially altering both the expression of housekeeping genes and genes involved in key biological processes. Antidepressants have been shown to influence the expression of genes related to neurotransmitter regulation, neuroplasticity, and inflammation, which are all critical in the pathophysiology of MDD—for example, drugs may upregulate genes involved in the serotonin and dopamine pathways as well as genes associated with synaptic plasticity and cellular stress responses. These changes in gene expression could contribute to the therapeutic effects of antidepressants and also complicate the interpretation of gene-based studies in MDD, highlighting the need for more detailed investigations into how these treatments modify gene expression. Clozapine, a second-generation antipsychotic (SGA), can significantly upregulate the expression of ELOVL3, CIDEA, CYC1, PGC1A, and TBX1 genes (21). The therapeutic drugs of psychiatric disorder can promote ATP5A1 expression in neuron-like cells and microglial cells (22). The main limitations of this study include the reliance on postmortem brain tissue, which may not fully capture the dynamic changes occurring in living patients, and the limited sample size from specific brain regions, potentially affecting the generalizability of the findings across the entire brain or to different populations. The findings from this study suggest that the identified hub genes (PARK2, CUL1, SKP1, CYC1, and ATP5A1) and the mitochondrial oxidative phosphorylation and ubiquitination-related biological pathways could serve as novel therapeutic targets for MDD.

## Data Availability

The datasets presented in this study can be found in online repositories. The names of the repository/repositories and accession number(s) can be found below: https://www.ncbi.nlm.nih.gov/, GSE80655.

## Supplementary material

Supplementary Figure 1 The optimal soft threshold of adjacency matrix calculation of gene co-expression network.
