# Supplementary figures and images for: A network analysis of gene co-expression in post-mortem brain tissues identifying novel genes and biological pathways underlying major depression
Source: Front Psychiatry. 2025 Jul 11;16:1556983. doi: 10.3389/fpsyt.2025.1556983 (PMC12289680; doi:10.3389/fpsyt.2025.1556983)

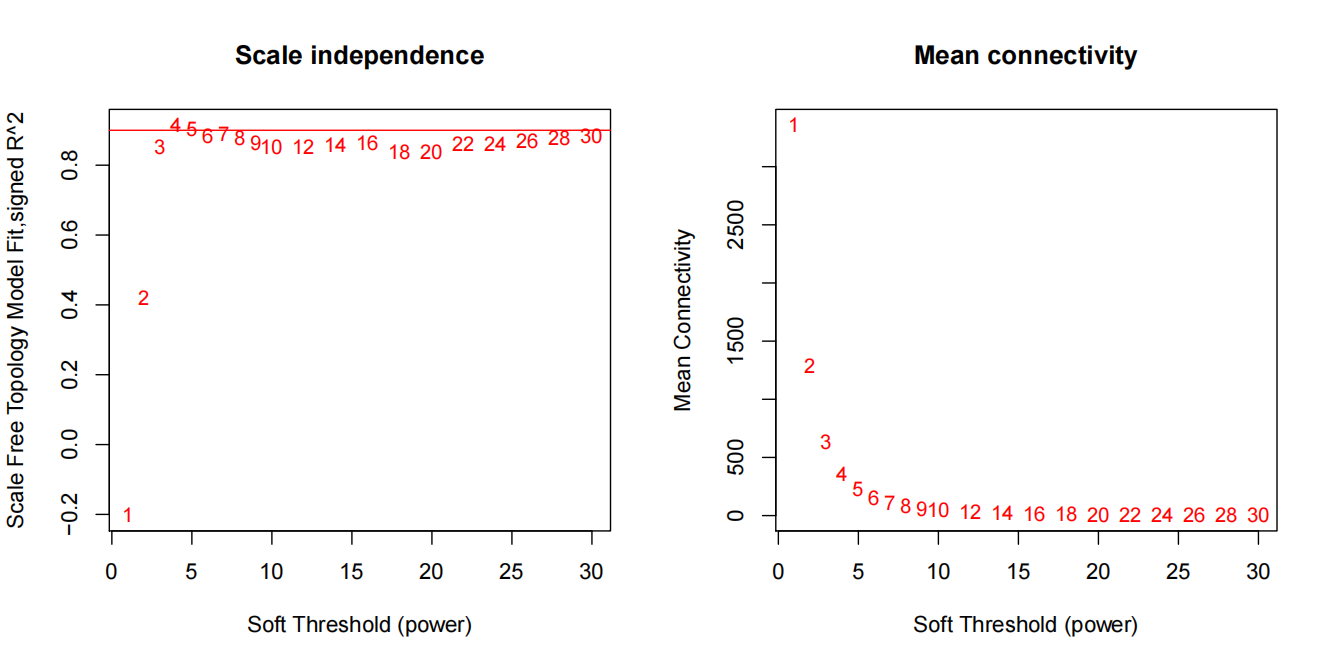

Supplement: Supplementary Figure 1 — The optimal soft threshold of adjacency matrix calculation of gene co-expression network. [file Image1.tif]
